# Differential Expression Profiles and Functional Prediction of tRNA-Derived Small RNAs in Rats After Traumatic Spinal Cord Injury

**DOI:** 10.3389/fnmol.2019.00326

**Published:** 2020-01-10

**Authors:** Chuan Qin, Hao Feng, Chao Zhang, Xin Zhang, Yi Liu, De-Gang Yang, Liang-Jie Du, Ying-Chun Sun, Ming-Liang Yang, Feng Gao, Jian-Jun Li

**Affiliations:** ^1^School of Rehabilitation Medicine, Capital Medical University, Beijing, China; ^2^Department of Spinal and Neural Functional Reconstruction, China Rehabilitation Research Center, Beijing, China; ^3^Center of Neural Injury and Repair, Beijing Institute for Brain Disorders, Beijing, China; ^4^Chinese Institute of Rehabilitation Science, Beijing, China; ^5^Beijing Key Laboratory of Neural Injury and Rehabilitation, Beijing, China; ^6^TCM Treatment Center, China Rehabilitation Research Center, Beijing, China

**Keywords:** tRNA-derived small RNA, spinal cord injury, sequencing, bioinformatics, BDNF

## Abstract

Spinal cord injury (SCI) is mostly caused by trauma. As the primary mechanical injury is unavoidable, a focus on the underlying molecular mechanisms of the SCI-induced secondary injury is necessary to develop promising treatments for patients with SCI. Transfer RNA-derived small RNA (tsRNA) is a novel class of short, non-coding RNA, possessing potential regulatory functions in various diseases. However, the functional roles of tsRNAs in traumatic SCI have not been determined yet. We used a combination of sequencing, quantitative reverse transcription-polymerase chain reaction (qRT-PCR), bioinformatics, and luciferase reporter assay to screen the expression profiles and identify the functional roles of tsRNAs after SCI. As a result, 297 differentially expressed tsRNAs were identified in rats’ spinal cord 1 day after contusion. Of those, 155 tsRNAs were significantly differentially expressed: 91 were significantly up-regulated, whereas 64 were significantly down-regulated after SCI (fold change > 1.5; *P* < 0.05). Bioinformatics analyses revealed candidate tsRNAs (tiRNA-Gly-GCC-001, tRF-Gly-GCC-012, tRF-Gly-GCC-013, and tRF-Gly-GCC-016) that might play regulatory roles through the mitogen-activated protein kinase (MAPK) and neurotrophin signaling pathways by targeting brain-derived neurotrophic factor (BDNF). We validated the candidate tsRNAs and found opposite trends in the expression levels of the tsRNAs and BDNF after SCI. Finally, tiRNA-Gly-GCC-001 was identified to target BDNF using the luciferase reporter assay. In summary, we found an altered tsRNA expression pattern and predicted tiRNA-Gly-GCC-001 might be involved in the MAPK and neurotrophin pathways by targeting the BDNF, thus regulating the post-SCI pathophysiologic processes. This study provides novel insights for future investigations to explore the mechanisms and therapeutic targets for SCI.

## Introduction

With the economic and social development, spinal cord injury (SCI) has become a growing public health concern worldwide. It is estimated that there are approximately 11,000 new patients with SCI in America each year, and currently, there are approximately 250,000 people with SCI (Qin et al., 2018; Hall et al., 2019; Liu et al., 2019). SCI is mostly caused by trauma, including the primary mechanical injury due to a rapid direct compression and contusion of the cord, and the subsequent secondary pathophysiological changes, such as edema, ischemia, apoptosis, autophagy, and oxidative stress, which usually lead to a permanent neurological impairment (Kang et al., 2018; Alizadeh et al., 2019; Vawda et al., 2019). The primary injury is in fact mostly unavoidable. Thus, there is an urgent need to attenuate the undesirable pathophysiological changes caused by the secondary injury in order to develop promising diagnostic and treatment methods for SCI in the clinical practice (Carelli et al., 2019).

However, the controversy about scientific evidence of neuroprotective and regenerative therapies for SCI has raged unabated for ages. Recently, extensive evidence related to the noncoding RNAs (ncRNAs), including long non-coding RNAs (lncRNAs; Zhang et al., 2018a,b), microRNAs (miRNAs; Liu et al., 2018), and circular RNAs (circRNAs; Qin et al., 2019), maybe at the point of breaking this impasse. The existing research has identified the critical role of these ncRNAs in alleviating the secondary pathophysiological damage after SCI (Jiang and Zhang, 2018; Wang et al., 2019).

Furthermore, with the help of next-generation sequencing techniques, many researchers found a heterogeneous population of small ncRNAs with lengths of 18–40 nucleotides cleaved from transfer RNA (tRNA), known as tRNA-derived small RNAs (tsRNAs; Lee et al., 2009; Li et al., 2018). Generally, there are two types of tsRNAs based on the length and cleavage sites of the tRNAs: tRNA-derived fragments (tRFs) and tRNA-derived stress-induced RNAs (tiRNAs). Interestingly, mounting evidence has shown that they are not merely byproducts of a random tRNA cleavage, but rather have critical functional roles as regulatory factors in the pathophysiologic processes of various diseases, such as tumor proliferation in ovarian cancer cells (Zhou et al., 2017) and cell cycle regulation in non-small cell lung cancer (Shao et al., 2017), and are potential biomarkers in prostate cancer (Olvedy et al., 2016). Nevertheless, to date, there are no studies focusing on the relationship between tsRNAs and traumatic SCI, and their molecular and intermolecular interactions and key signaling pathways remain to be elucidated. In addition, there is a growing body of evidence that various tsRNAs are generated under stress conditions and act as miRNAs in gene expression regulation by targeting the messenger RNAs (mRNAs; Kumar et al., 2014; Gebetsberger et al., 2017). As a result, it is rational to speculate that tsRNAs may be involved in the progression of the pathophysiological changes after SCI.

**Figure 1:**
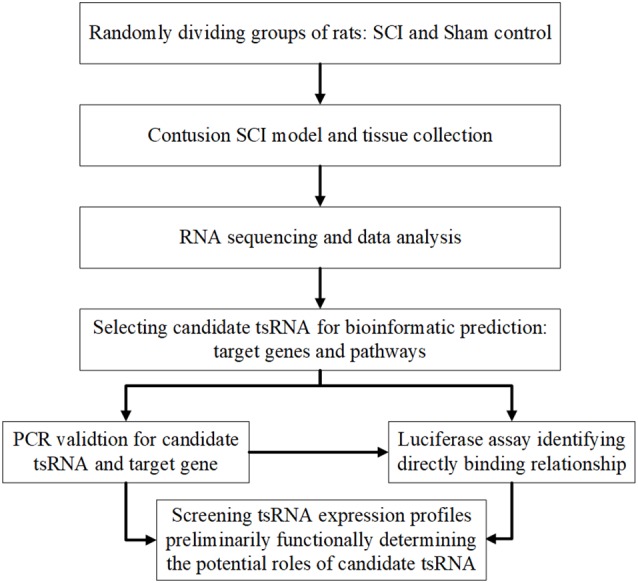
Study design illustration. SCI, spinal cord injury; FC, fold change; qRT-PCR, quantitative real-time polymerase chain reaction.

Therefore, the aim of this investigation was to explore the tsRNAs’ expression profiles and preliminarily determine the potential functional roles of candidate tsRNAs in the pathophysiology after traumatic SCI. The study design is presented in Figure 1. These findings may provide new clues for studying the mechanisms underlying SCI and novel molecular targets for the clinical therapy of SCI.

In our study, we explored the relationship between tsRNAs and SCI. First, we examined the expression profiles of tsRNAs in rats’ spinal cords after SCI using next-generation sequencing. Second, bioinformatics methods were used to perform tsRNA/mRNA interaction networks and predict the potential role of the target genes of chosen tsRNAs in the process after SCI. Third, four candidate tsRNAs and their potential target mRNAs were further validated by quantitative reverse transcription-polymerase chain reaction (qRT-PCR) to reveal their trends of expression level alteration after SCI. Finally, we used the luciferase reporter assay to detect the direct combination between the selected tsRNA and its target gene.

## Materials and Methods

### Animals and Experimental Groups

This study was performed in accordance with the principles of the Basel Declaration and the recommendations of the National Institute of Health Guide for the Care and Use of Laboratory Animals (NIH Publications No. 8023, revised 1978). The protocol was approved by the Institutional Animal Care and Use Committee of Capital Medical University. Male Sprague–Dawley rats (250–300 g) were housed for at least 7 days before the study in a temperature- (22–25°C) and humidity-controlled (50% relative humidity) animal facility with 12-h light/dark cycles. The animals had free access to food and water. However, food was withheld overnight before surgery.

Similar as in our previous study (Qin et al., 2019), 12 rats were randomly assigned into two groups using a computer-generated randomization schedule: the rats in the sham control group (*n* = 6) underwent laminectomy alone without contusion, and the rats in the SCI group (*n* = 6) underwent laminectomy and were subjected to contusion. The operators performing the surgeries were blinded to the experimental groups. Before and after surgery, our two experienced researchers performed a behavioral test of the rats in the two groups using the Basso, Beattie, and Bresnahan (BBB) score, frequently used to access the locomotor function in rats (Scheff et al., 2002). The BBB score ranges from 0 (no hindlimb movement) to 21 (normal movement), and the final score was given by a consensus.

### Contusion SCI Model and Tissue Collection

In this study, the contusion injury method was used to induce moderate injuries in the rat models, similar to our previous study (Qin et al., 2019). In brief, all rats were anesthetized by an intraperitoneal injection of sodium pentobarbital solution at 40 mg/Kg body weight, and then their backs were shaved and sterilized. After holding or fixing the rats in a stereotaxic frame, a 4-cm longitudinal midline incision was made to expose the spinous processes of the thoracic (T) 9–T11 vertebrae. Following stripping of the paraspinal muscles, laminectomy was performed at the T10 level to expose the spinal dura mater without tearing it in a sterile condition. Subsequently, the rats were clamped by the T9 and T11 spinous processes using a sterile forceps, followed by a spinal cord contusion induced by an Infinite Horizon Impactor (IH-0400 Impactor, Precision Systems and Instrumentation, LLC., Natick, MA, USA), causing a moderate injury at the T10 level. Regarding the parameter settings, a standard rat tip impactor size (2.5 mm in diameter) and programmable force levels (225 kDynes) were applied, which were similar with the NYU weight-drop device settings (2.5 mm in diameter, 10 g rod, height of 12.5 mm) according to the conversion equation (Khuyagbaatar et al., 2015). Errors greater than 3% in terms of force levels and tears of the spinal dura mater were not accepted.

After inducing contusions, the operative region was gently washed twice with a warm 0.9% normal saline (37°C, 2 ml) to avoid local infection. The cord surface showed signs of a subarachnoid hematoma and an intense dark brown/purple color. The rats with swinging tails that quickly retracted their lower limbs immediately after the SCI were regarded as eligible, as previously described (Hu et al., 2015). Finally, the wound was sutured in layers. A warm environment was established to maintain body temperature during the surgery. Notably, 4 ml of Ringer lactate solution was administered intraperitoneally for electrolyte and fluid supplementation. Penicillin (40,000 U) was administered by intramuscular injection to prevent a systemic infection. The rats were then housed in individual cages, and their bladders were emptied manually every 8 h until they were sacrificed.

At 24 h after surgery, the rats in all groups were euthanized with an overdose of 40 mg/kg sodium pentobarbital solution, and a 1-cm long segment of the spinal cord, including the injury epicenter, was quickly dissected and collected without a prior transcardial perfusion and was fresh frozen in liquid nitrogen to prevent RNA degradation, similarly to a previously reported method (Pang et al., 2016).

### RNA Extraction and Quality Control

In brief, the total RNA was isolated using TRIzol reagent (Invitrogen, Carlsbad, CA, USA) based on the manufacturer’s instructions. Before the sequencing experiment, the integrity and quantity of each RNA sample were checked using agarose gel electrophoresis and the NanoDrop ND-1000 (NanoDrop, Wilmington, DE, USA) instrument.

### Pretreatment of tsRNA and Library Preparation

In order to prevent RNA modifications that interfere with the small RNA-seq library construction, we performed the following treatments before the library preparation for the total RNA samples: 3′-aminoacyl (charged) deacylation to 3′-OH for 3′-adaptor ligation, 3′-cP (2′,3′-cyclic phosphate) removal to 3′-OH for 3′-adaptor ligation, 5′-OH (hydroxyl group) phosphorylation to 5′-P for 5′-adaptor ligation, and m1A and m3C demethylation for efficient reverse transcription. Subsequently, sequencing libraries were size-selected for the RNA biotypes to be sequenced using an automated gel cutter. The sequencing library was determined by the Agilent 2100 Bioanalyzer using the Agilent DNA 1000 chip kit (Agilent, part # 5067-1504).

### Libraries Denaturation and Sequencing

The libraries were denatured and diluted to a loading volume of 1.3 ml and a loading concentration of 1.8 pM. The diluted libraries were loaded onto a reagent cartridge and forwarded to sequencing, which was performed on an Illumina NextSeq 500 system using a NextSeq 500/550 V2 kit (#FC-404-2005, Illumina, San Diego, CA, USA), according to the manufacturer’s instructions. The sequencing was performed by running 50 cycles.

### Data Collection and Analysis

The raw sequencing data that passed the Illumina chastity filter were used for the following analysis. After the Illumina quality control, the sequencing reads were 5′,3’-adaptor trimmed and filtered for over 15 nucleotides by the Cutadapt software. Using the NovoAlign software (v2.07.11), the trimmed reads were aligned to the mature tRNA sequences, allowing for one mismatch only, and the reads that did not map were aligned to the precursor tRNA sequences, allowing for one mismatch only, using the BowTie software (Langmead et al., 2009).

The differentially expressed tsRNAs were screened based on the count value using R package edgeR (Qin et al., 2019). Moreover, the tsRNA expression levels could be measured and normalized as counts per million (CPMs) of the total aligned tRNA reads. The tsRNA expression profiling and differential expression analysis were calculated by the average CPM. When comparing the two groups for profile differences (SCI group vs. sham group), the “fold change (FC),” i.e., the ratio of the group averages between the groups was computed for each tsRNA. An FC > 1.5 and a *P*-value < 0.05 were considered as a significantly different expression, and the tsRNAs with such values were chosen for the next analysis. Principal component analysis (PCA), correlation analysis, pie plots, Venn plots, hierarchical clustering, scatter plots, and volcano plots were performed in an R or a Perl environment for statistical computing and graphical presentation of the expressed tsRNAs.

### Bioinformatic Prediction

The four significantly differentially expressed tsRNAs (FC > 1.5 and *P*-value < 0.05) selected from the sequencing data were analyzed using bioinformatic methods. The candidate selection was based on several points: (1) higher FC and lower *P*-value; (2) higher CPM in both groups and balanced expression of each rat in each group; and (3) Referring to previous publications to achieve more evidence of similar subtype of tsRNA.

First, in terms of target prediction of the candidate tsRNAs, the tsRNAs contained some seed sequences that could match the crosslink-centered regions of the target mRNAs (Kim et al., 2017). Mounting evidence has strongly suggested that tsRNAs could act as miRNAs, silencing the target mRNA *via* complementary base pairing (Kumar et al., 2014). According to a previous study (Li et al., 2019), three common algorithms were used to predict the tsRNA targets, including TargetScan^1^, miRanda^2^, and miRDB^3^. Notably, to reduce the false-positive results, only the genes predicted by all three software were considered as target mRNAs of the tsRNAs. A graph of the tsRNA/mRNA network was derived using the Cytoscape software (version 3.5.1, the Cytoscape Consortium, San Diego, CA, USA) to visualize these relationships.

Second, to assign the biological annotation of the targets, pathway, and process enrichment analyses were performed. We used Gene Ontology (GO^4^) to reveal the biological process, cellular component, and molecular function of the target mRNAs. Significant pathways were identified using the pathways in the Kyoto Encyclopedia of Genes and Genomes database (KEGG^5^). A *P* < 0.05 indicated the significance of the GO and KEGG pathway terms. The false discovery rate was calculated to correct the *P*-values. More specifically, *P*-values were calculated based on an accumulative hypergeometric distribution and the enrichment factor was the ratio between the observed count and the count expected by chance.

### qRT-PCR Assay

The expression of the four tsRNAs selected through the bioinformatic analysis and that of the target mRNAs was validated using a qRT-PCR assay. The cord tissue samples from the rats in the SCI (*n* = 6) and sham groups (*n* = 6), including the tissue collected for the sequencing (*n* = 4 in both groups), as well as additional tissues (*n* = 2 in both groups) were used in the PCR experiment. As previously reported (Zhang et al., 2018), the total RNA was isolated using TRIzol reagent according to the manufacturer’s standard protocols (Invitrogen, Carlsbad, CA, USA). RNA quantification and quality were assessed using NanoDrop ND-1,000, and RNA integrity was verified by electrophoresis on a denaturing agarose gel. Next, we synthesized complementary DNA (cDNA) in line with the manufacturer’s instructions. qRT-PCR was performed in a ViiA 7 Real-time PCR System (Applied Biosystems, Foster City, CA, USA) using a PCR master mix (2×, Arraystar). The parameter settings were as follows: 95°C denaturation (10 min), 95°C (10 s), and 60°C (60 s), which was repeated for 40 cycles. After the amplification reaction was finished, the procedure was performed as follows: 95°C (10 s), 60°C (60 s), and 95°C (15 s). The relative tsRNA expression levels were calculated using the relative standard curve method (Larionov et al., 2005) and were normalized to U6 and β-actin, as endogenous. All reactions were performed in triplicate.

### Luciferase Assay

Luciferase constructs containing either base pairs of the brain-derived neurotrophic factor (BDNF) 3′ UTR or two base-pair regions containing the predicted binding sites and a second construct, containing site-directed mutations in the predicted binding site, were prepared using methods as previously described (Arcaroli et al., 2012). The BDNF 3′ UTR, including the predicted binding sites for tiRNA-Gly-GCC-001, was cloned into a psiCheck-2 dual-luciferase vector. Mutant BDNF 3′ UTR luciferase vectors were produced in the predicted tiRNA-Gly-GCC-001-binding regions. These plasmids and a tiRNA-Gly-GCC-001 mimic were co-transfected into HEK293T cells. Finally, luciferase assays were conducted using the dual-luciferase system (Promega, Madison, WI, USA), according to the manufacturer’s instructions.

**Table 1:** The top 10 up-regulated and down-regulated tsRNAs ranked by fold changes after SCI.

tsRNA	Type	Length	Fold change	*P*-value	Regulation
tiRNA-Asn-GTT-001	tiRNA-5	33	159.003645915166	1.41E-19	Up
tRF-Met-CAT-017	tRF-5c	30	122.01571606758	2.45E-15	Up
tRF-Met-CAT-051	tRF-5c	28	105.133667532338	5.65E-16	Up
tRF-Ser-AGA-016	tRF-5b	23	85.3172694348191	1.94E-40	Up
tRF-Met-CAT-016	tRF-5c	29	77.9150421396709	2.30E-15	Up
tRF-Gln-CTG-005	tRF-2	14	75.0513113045871	1.05E-20	Up
tRF-Ser-AGA-017	tRF-5b	24	57.6418498950225	2.52E-38	Up
tiRNA-Met-CAT-002	tiRNA-5	31	40.6199926628678	7.39E-14	Up
tRF-His-GTG-017	tRF-2	14	40.6053037913958	4.27E-18	Up
tRF-Asn-GTT-047	tRF-5c	32	33.3246942968211	2.66E-13	Up
tRF-Thr-AGT-022	tRF-3b	22	0.222109257777734	1.07E-05	Down
tRF-Ala-AGC-057	tRF-3b	22	0.243965889353342	1.06E-06	Down
tRF-Cys-GCA-007	tRF-3a	18	0.246922741938728	4.34E-07	Down
tRF-Ala-TGC-027	tRF-3b	22	0.274660579014769	3.43E-06	Down
tRF-Ala-TGC-035	tRF-3b	22	0.291169582918833	3.24E-04	Down
tRF-Ala-AGC-056	tRF-3b	22	0.293223504647025	4.91E-06	Down
tRF-Ala-TGC-002	tRF-3b	19	0.301367786961553	2.09E-05	Down
tRF-Leu-TAG-014	tRF-3a	17	0.30328867954121	1.77E-06	Down
tRF-Leu-CAG-023	tRF-3b	22	0.318608293880152	4.18E-06	Down
tRF-Cys-GCA-029	tRF-3a	17	0.32652518025552	8.72E-05	Down
**The candidate tsRNAs selected for bioinformatics and PCR**					
tiRNA-Gly-GCC-001	tiRNA-5	33	6.41547526797363	7.46E-13	Up
tRF-Gly-GCC-012	tRF-5c	28	6.05230928162583	4.36E-12	Up
tRF-Gly-GCC-013	tRF-5c	29	3.57949960289882	1.15E-06	Up
tRF-Gly-GCC-016	tRF-5c	32	3.35515175746991	4.78E-07	Up

### Statistical Analysis

Statistical analysis was performed using SPSS software (version 21.0, Chicago, IL, USA). The results are shown as the mean ± standard error of the mean (SEM). Student’s *t*-test was used to compare the significant differences between the two groups. The level of significance was set at *P* < 0.05.

### Database and Accession Numbers

The raw data of the tsRNA-Seq in our study were deposited at the NCBI Gene Expression Omnibus (GEO) under the accession number GSE133157.

## Results

### Altered Expression Profiles of tsRNAs in Spinal Cord Tissues After SCI

Prior to injury, the BBB scores of all rats were 21. The rats in the sham group maintained a score of 21 during the experimental period, indicating the integrity of the spinal cord. However, in the SCI group, no hindlimb motor performance was observed immediately after recovery from anesthesia and before sacrifice to reduce the contusion error and heterogeneity, indicating a loss of the locomotive function due to the contusion.

**Figure 2:**
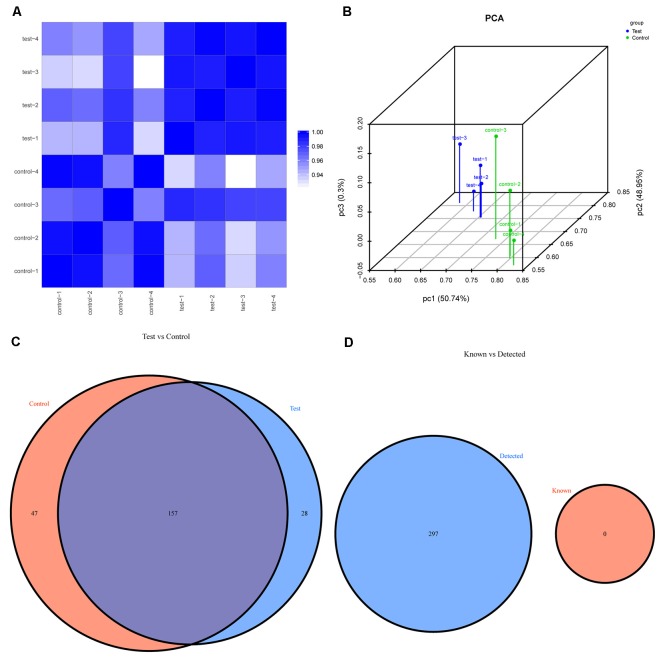
The expression level analysis. **(A)** Heat-map of the correlation coefficient from all samples. The color in the panel represents the correlation coefficient of the two samples. The blue represents the two samples with a high correlation coefficient, and the white represents the low similarity of the two samples.** (B)** Primary component analysis: X, Y and Z axes represent the three main factors that affected the expression level of the sample. The colored point represents the corresponding sample, and its location shows the main character of the sample. The space distance represents the similarity of the data size.** (C)** Venn diagram based on the number of commonly expressed and specifically expressed tsRNAs. This diagram shows the number of tsRNAs which were expressed in both groups and also indicated the number of the specifically expressed tsRNAs.** (D)** Venn diagram based on the number of tsRNAs known and detected. This diagram shows the number of tsRNAs detected in this project and collected in the tRFdb.

The first set of questions aimed to identify the tsRNA expression levels in the two groups; thus, the tsRNA-Seq analysis was used. Prior to analysis, sequencing quality control was performed *via* the quality score plot of each sample (see Supplementary Figures S1, S2, and Supplementary Table S1). A *Q* score above 30 (>99.9% correct) indicated high-quality data and a very large proportion of the bases in each sample achieved a *Q* ≥ 30 (all more than 92%). After the quality filtering, according to the expression level of each sample, we calculated the correlation coefficient between any two of the samples, which is an important evaluation criterion of the reliability and reasonability of the sample selection, showing that the two compared samples were quite similar (see Figure 2A). In addition, we used PCA, a statistical method used for an unsupervised analysis to reduce the dimension of large data sets, and it was a useful tool to explore the sample classes based on the expression, showing a distinguishable tsRNA expression profiling among eight samples (see Figure 2B).

**Figure 3:**
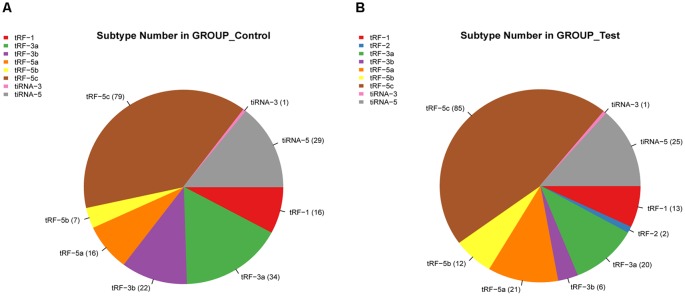
Pie chart of each tsRNA subtype. **(A)** Pie chart of the distribution of tsRNA subtypes in the sham group. **(B)** Pie chart of the distribution of tsRNA subtypes in the SCI group.

**Figure 4:**
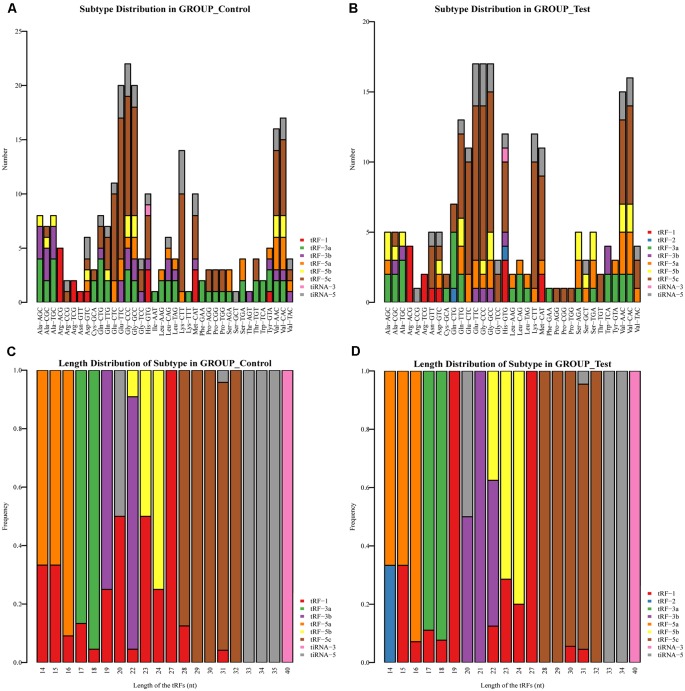
Stacked Bar Chart. **(A)** The number of tsRNA subtypes against tRNA isodecoders in the sham group. **(B)** The number of tsRNA subtypes against tRNA isodecoders in the SCI group. **(C)** The Frequency of Subtype against Length of the tsRNAs in the sham group. **(D)** The Frequency of Subtype against Length of the tsRNAs in the SCI group.

We identified a dysregulated expression of all 297 tsRNAs between the SCI and sham groups. In Figure 2C, the Venn diagram presents a total of 157 commonly expressed tsRNAs, 47 tsRNAs specifically expressed in the sham group, and 28 tsRNAs specifically expressed in the SCI group. In Figure 2D, the Venn diagram shows that all 297 dysregulated tsRNAs in this study were known tRFs from tRFdb. Next, as shown in Figures 3A,B, a pie chart was performed of each tsRNA subtype, indicating that most tsRNAs were generated from mature tRNAs (subtypes of tsRNAs, except for tRF-1). In those tsRNAs, the expression levels of each tsRNA subtype were quite different. Overall, the expression level of tRF-5 s was increased, whereas that of other subtypes was decreased after the SCI, as compared to the sham group. Furthermore, the number of tsRNA subtypes against tRNA isodecoders and the frequency of subtypes against the length of the tsRNA were compared *via* the stacked bar charts (see Figures 4A–D).

**Figure 5:**
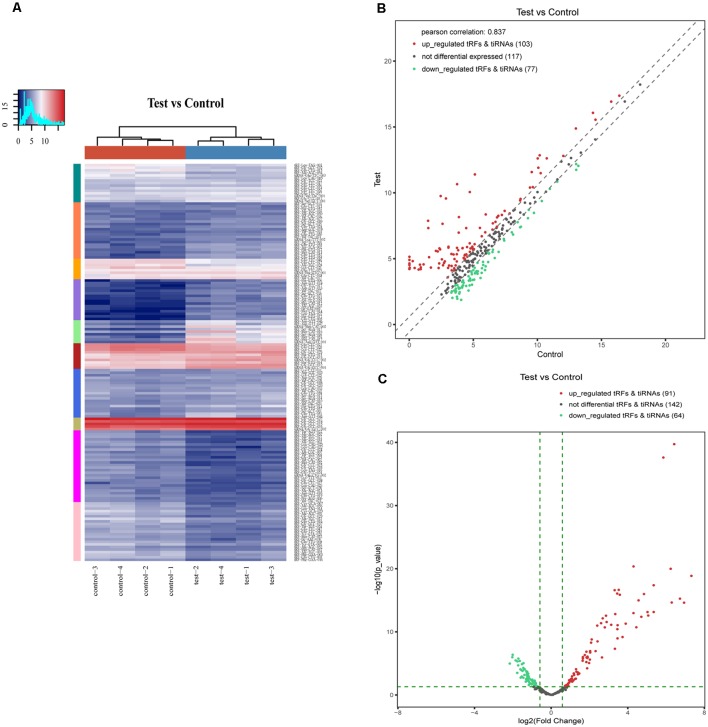
Hierarchical clustering and difference in the tsRNA expression between the two groups. **(A)** The hierarchical clustering heat-map for the tsRNAs. The color in the panel represents the relative expression level (log2-transformed). The colored bar top at the top panel showed the sample groups, and the colored bar at the right side of the panel indicated the divisions that were performed using K-means. **(B)** The scatter plots of differentially expressed tsRNAs. tsRNAs above the top line (red dots, up-regulation) or below the bottom line (green dots, down-regulation) indicate more than 1.5-fold change between the two compared groups. Gray dots indicate non-differentially expressed tsRNAs.** (C)** The volcano plots of differentially expressed tsRNA. The values of the X and Y axes in the volcano plot are log2 transformed fold change and −log10 transformed *P*-values between the two groups, respectively. Red/Green circles indicate statistically significant differentially expressed tsRNAs with a fold change of no less than 1.5 and a *P*-value ≤ 0.05 (Red: up-regulated; Green: down-regulated). Gray circles indicate non-differentially expressed tsRNA, with FC and/or *q*-value that are not meeting the cut off thresholds.

In addition, we identified that a total of 155 tsRNAs were significantly differentially expressed between the two groups: 91 were significantly up-regulated, whereas 64 were significantly down-regulated (SCI vs. sham; FC > 1.5; *P* < 0.05). The experimental data on the top 10 upregulated and downregulated tsRNAs ranked by fold changes are presented in Table 1. As shown in Figure 5A, hierarchical clustering was performed using the significantly differentially expressed tsRNAs, indicating a distinguishable tsRNA expression profiling among the samples. Next, scatter plots were created presenting the tsRNA expression variation (or reproducibility) between the SCI and sham control groups using the fold change (Figure 5B) and volcano plots were constructed using the fold change values and *P*-values to visualize the differential expression between the two different conditions (Figure 5C).

### Bioinformatic Prediction

**Figure 6:**
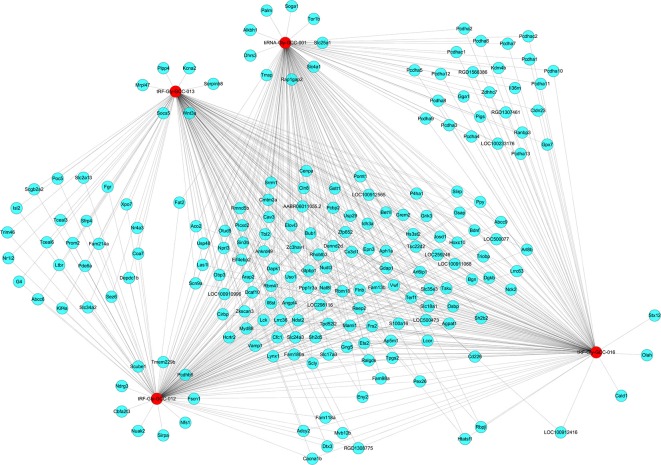
The tsRNA/mRNA network analysis. The network included the four candidate tsRNAs and their predicted target mRNAs (Nodes in red color are tsRNAs; nodes in light-blue color are mRNAs).

The four significantly differentially expressed tsRNAs, namely, tiRNA-Gly-GCC-001, tRF-Gly-GCC-012, tRF-Gly-GCC-013, and tRF-Gly-GCC-016, selected from the tsRNAs commonly expressed in both groups, were subsequently analyzed using bioinformatic tools. First, based on the knowledge that the tsRNAs could perform an miRNA-like mode of action, that is, recognize their mRNA targets using their seed sequence (positions 2–7nt at their 5′ ends) and inhibit the global mRNA translational activities (Kumar et al., 2014), three kinds of algorithms were used to predict the mRNA targets of the four candidate tsRNAs. As a result, we constructed tsRNA/mRNA interaction networks using the top 150 target mRNAs of each tsRNA based on the prediction score (Figure 6).

**Figure 7:**
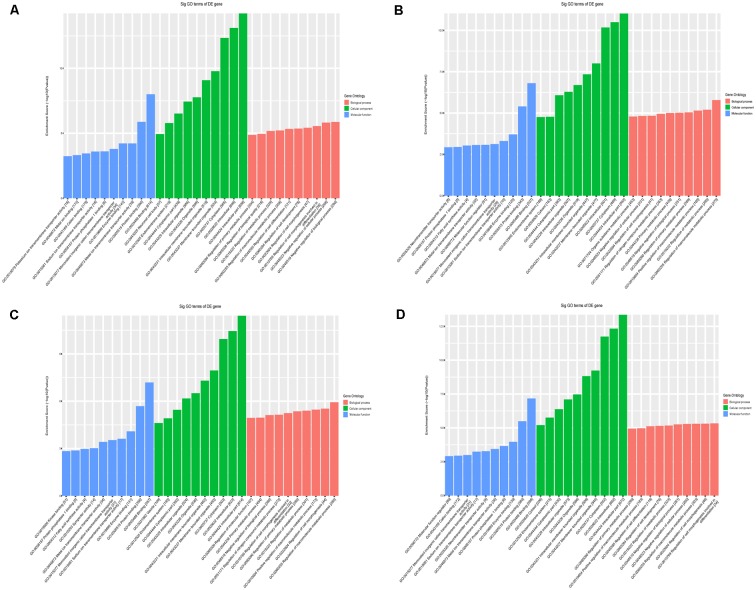
The general GO annotations for cellular component, molecular function, and biological processes of the target mRNAs regulated by the four candidate tsRNAs. **(A)** tiRNA-Gly-GCC-001. **(B)** tRF-Gly-GCC-012. **(C)** tRF-Gly-GCC-013. **(D)** tRF-Gly-GCC-016.

Second, we conducted a bioinformatic analysis of the functions of all target mRNAs of each candidate tsRNA using the KEGG pathway and GO biological processes. GO bioinformatic analysis covers three domains: biological process, cellular component, and molecular function. For each part, we performed a classification for the significantly enriched terms of each candidate tsRNA—a top 10 enrichment score with counts and *P*-values. In terms of cellular component and molecular function (Figure 7), the general results showed that the most significant enrichment and the most meaningful terms were binding (GO:0005488) and intracellular part (GO:0044424) in each of the four tsRNA predictions (Supplementary Figures S3, S4), suggesting a functional role of the target genes of the candidate tsRNAs at the molecular and cellular level. However, the major biological processes surveyed by GO were diverse in the four tsRNA predictions, namely, negative regulation (GO:0048519), regulation of the macromolecular metabolic process (GO:0060255), and regulation of the cellular morphogenesis, involved in differentiation (GO:0010769), suggesting their potential roles in the biological processes (Supplementary Figure S5).

**Figure 8:**
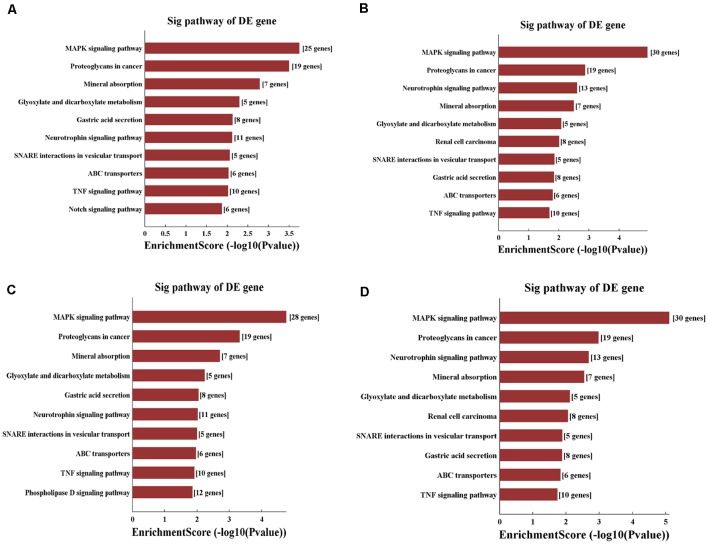
KEGG pathway analysis of the target mRNAs regulated by the four candidate tsRNAs. **(A)** The bar plot shows the top 10 enrichment score values of the significantly enriched pathway for tiRNA-Gly-GCC-001. **(B)** The bar plot shows the top 10 enrichment score values of the significantly enriched pathway for tRF-Gly-GCC-012. **(C)** The bar plot shows the top 10 enrichment score values of the significantly enriched pathway for tRF-Gly-GCC-013.** (D)** The bar plot shows the top 10 enrichment score values of the significantly enriched pathway for tRF-Gly-GCC-016.

According to the KEGG enrichment analysis, mitogen-activated protein kinase (MAPK) signaling pathway (rno04010), proteoglycans in cancer (rno05205), and neurotrophin signaling pathway (rno04722) were significantly identified in general view for each candidate tsRNA (Figure 8). In addition, in view of the prediction score of the target mRNAs and previous publications, it is plausible to speculate that these four tsRNAs might be involved in the regulation of the biological processes *via* the MAPK and neurotrophin signaling pathway. All tsRNA-targeted genes in both pathways are shown in Supplementary Figures S6, S7. Notably, BDNF is one target mRNA of the four significantly differentially expressed tsRNAs as well as a vital gene involving in MAPK and neurotrophin signaling pathway (binding sites were shown in Supplementary Figure S8). In addition, emerging evidence has suggested that BDNF, a substance that supports the growth and maintenance of brain cells, is closely related to the regulation of the pathophysiological processes in the acute phase of SCI, such as to attenuate the secondary injury (Keefe et al., 2017; Zhang et al., 2018a,b; Li et al., 2019). Therefore, we reasonably speculate that tiRNA-Gly-GCC-001, tRF-Gly-GCC-012, tRF-Gly-GCC-013, and tRF-Gly-GCC-016 might regulate the BDNF and thus play a vital role in the pathophysiological process *via* the MAPK and neurotrophin signaling pathway.

### Verification of qRT-PCR

**Figure 9:**
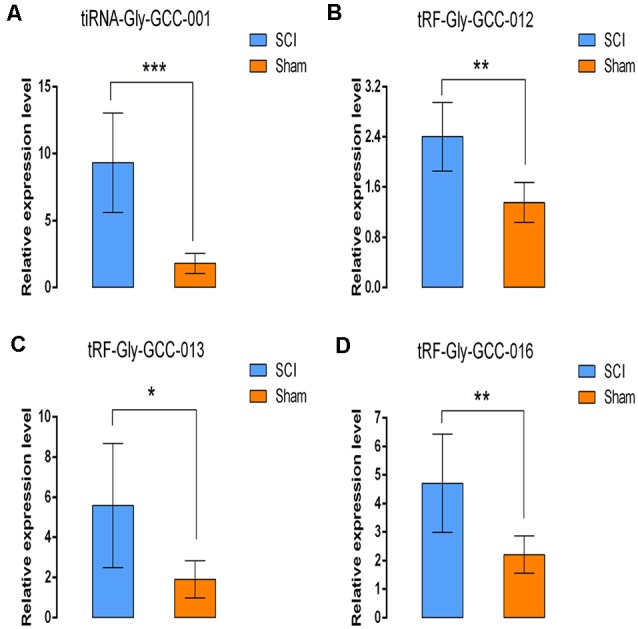
Validation of the four selected tsRNAs using qRT-PCR. Compared with the sham control, tiRNA-Gly-GCC-001, tRF-Gly-GCC-012, tRF-Gly-GCC-013, tRF-Gly-GCC-016 were all significantly upregulated in the SCI group **(A–D)**. The data were normalized using the mean ± standard error of the mean (SEM; *n* = 6 per group). *P < 0.05, **P < 0.01, ***P < 0.001.

In this section, our aim was to validate the tsRNA-Seq results *via* detecting the expression level of the four candidate tsRNAs using a low-throughput qRT-PCR. As a result, compared with the sham control, tiRNA-Gly-GCC-001, tRF-Gly-GCC-012, tRF-Gly-GCC-013, and tRF-Gly-GCC-016 were all significantly up-regulated in the SCI group, showing a similar expression pattern in both the sequencing and PCR data, see Figures 9A–D. The fold change and *P*-values of each candidate tsRNA between the groups in terms of sequencing and PCR analysis are presented in Table 2, to make data interpretation more straightforward. The sequences of the primers used for the qRT-PCR are listed in Table 3. In consideration of the FC and *P*-values, tiRNA-Gly-GCC-001 was selected for the next binding experiment.

### The Relationship of tiRNA-Gly-GCC-001 and BDNF

**Figure 10:**
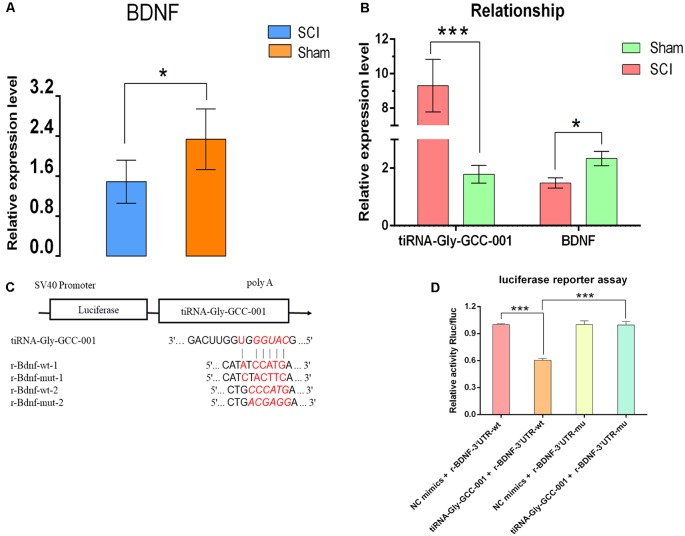
The relationship of tiRNA-Gly-GCC-001 and brain-derived neurotrophic factor (BDNF) validated by PCR and luciferase reporter assay. **(A)** The BDNF expression in the SCI group was significantly downregulated compared with the sham control (*n* = 6 per group). *p < 0.05.** (B)** An opposite change of expression between tiRNA-Gly-GCC-001 and BDNF was identified after injury, indicating that BDNF might act as a target gene inhibited by tiRNA-Gly-GCC-001. **(C)** A schematic of the base pair regions of the BDNF 3′ UTR and the predicted binding site for tiRNA-Gly-GCC-001 is shown with the wild type and mutant seed sequences listed below. **(D)** The repressive effect of tiRNA-Gly-GCC-001 on the activity of the site of BDNF 3′UTR measured by luciferase reporter assay, indicating that tiRNA-Gly-GCC-001 directly targeted BDNF. ***p < 0.001.

To identify their relationship, low-throughput qRT-PCR for BDNF and luciferase assay were performed. As a result, BDNF was found to be significantly down-regulated after SCI, indicating an opposite change of expression after injury compared to that of the candidate tsRNA (see Figures 10A,B), which suggested that BDNF might act as a target gene negatively regulated by tiRNA-Gly-GCC-001. In terms of the luciferase reporter assay, a sequence alignment of the tiRNA-Gly-GCC-001 with the 3′UTR of the BDNF was shown and the BDNF expression was inhibited by the tiRNA-Gly-GCC-001 directly targeting its 3′UTR (Figures 10C,D). This lays the foundation for our future research of the mechanism of this process.

## Discussion

In summary, we initially revealed the expression profiles of the tsRNAs using RNA-sequencing. Second, bioinformatic analysis prompted that four tsRNAs might inhibit the BDNF, thereby regulating the pathophysiological process *via* the MAPK and neurotrophin signaling pathway. Next, using PCR, we validated the candidate tsRNAs and found the opposite trends of expression levels of the tsRNAs and BDNF in the SCI vs. the sham group. Finally, we identified tiRNA-Gly-GCC-001 directly targeted the BDNF using the luciferase reporter assay. Taken together, the striking observations from our study indicate that these candidate tsRNAs might be involved in the pathophysiologic processes after traumatic SCI. In reviewing the literature, the present study, to the best of our knowledge, offers the first comprehensive assessment of the tsRNAs expression patterns and subsequent functional prediction and preliminarily target validation after traumatic SCI, providing strong evidence for further investigation.

Most of the human genome is composed of ncRNAs, which are widely involved in physiological and pathological activities, and are closely related to many diseases (Chen et al., 2019). These universal nucleic acid entities are commonly considered as a special type of RNA that cannot be translated into proteins (Yang et al., 2019). tsRNAs are the most common type of small ncRNAs, a recently rising star of the ncRNAs, that are reported to participate in the regulation of RNA processing and protein translation (Ivanov et al., 2011). Interestingly, tsRNAs, a heterogeneous population of small ncRNAs with lengths of 18–40 nucleotides, were first considered to be the byproducts of random tRNA cleavage. Generally, tsRNAs include two main types based on the length and cleavage sites of the tRNAs: tRFs and tiRNAs. Mostly, they are generated under stress conditions (Lee and Collins, 2005).

Growing evidence has shown that tsRNAs are implicated in various biological processes and human illness, such as tumors, cardiovascular system diseases, epigenetics, and neurological diseases, that scientists are increasingly concerned with, in the recent years (Olvedy et al., 2016; Zhou et al., 2017; Elkordy et al., 2018; Zhang et al., 2018). In terms of the functional roles of the tsRNAs in neurodegeneration, as an example, tiRNAAla and tiRNACys and their DNA analogs with a G4 motif could promote neuronal survival under stress conditions and provide new chances for the treatment of neurodegenerative disorders in the future (Li et al., 2018). In addition, the transfection of a particular tRF-5 into patient-derived neurons resulted in a reduction in cell survival under oxidative stress *in vitro* (Schaffer et al., 2014). In terms of neurotrauma, Elkordy et al. (2018, 2019) found that in PC-12, the rat neuronal cell line, tRNA cleavage and tiRNA generation were initiated under stress conditions induced by arsenite and hydrogen peroxide or ischemic-reperfusion. Notably, they identified that the generation of tiRNAs occurred prior to severe cell damage, indicating their potential roles as novel biomarkers for cell damage assessment. However, no previous studies have profiled the tsRNA expression in the spinal cord after SCI. As a result, after sequencing and PCR validation, the striking results in our study indicated that the expression levels of tsRNAs were significantly altered in rats’ spinal cord after SCI compared to the sham controls.

**Table 2:** Comparison for candidate tsRNAs expression in sequencing and PCR (SCI vs. Sham; FC, fold change).

tsRNAs	Sequencing			PCR		
	FC	*P*-value	Regulation	FC	*P*-value	Regulation
tiRNA-Gly-GCC-001	6.42	7.46E-13	Up	5.21	0.0007	Up
tRF-Gly-GCC-012	6.05	4.36E-12	Up	1.77	0.0023	Up
tRF-Gly-GCC-013	3.58	1.15E-06	Up	2.95	0.0187	Up
tRF-Gly-GCC-016	3.36	4.79E-07	Up	2.13	0.0076	Up

**Table 3:** Sequences of primers used for qRT-PCR assay.

Gene name	Primer sequence	Ta Opt (°C)	Product size(bp)
U6	F:5′ GCTTCGGCAGCACATATACTAAAAT 3′ R:5′ CGCTTCACGAATTTGCGTGTCAT 3′	60	89
β-actin	F:5′ CGAGTACAACCTTCTTGCAGC 3′ R :5′ ACCCATACCCACCATCACAC 3′	60	202
tiRNA-Gly-GCC-001	F:5′ GCATGGGTGGTTCAGTGGTAG 3′ R:5′ ACGTGTGCTCTTCCGATCTCA 3′	60	52
tRF-Gly-GCC-012	F:5′ GCATGGGTGGTTCAGTGGTAG 3′ R:5′ CGTGTGCTCTTCCGATCTGA 3′	60	46
tRF-Gly-GCC-013	F:5′ GCATGGGTGGTTCAGTGGTAG 3′ R:5′ CGTGTGCTCTTCCGATCTCG 3′	60	47
tRF-Gly-GCC-016	F:5′ GCATGGGTGGTTCAGTGGTAG 3′ R:5′ ACGTGTGCTCTTCCGATCTAGG 3′	60	51
BDNF	F:5′ GCGTGTGTGACAGTATTAGCGAG 3′ R:5′ GGCATTGCGAGTTCCAGTG 3′	60	210

In terms of the functions of the tsRNAs, an increasing number of tRFs and tiRNAs have been reported to act as functional regulatory factors in physiological processes and the cellular metabolism, indicating their non-random processing. Namely, tsRNAs have been confirmed to act as miRNA in gene expression regulation (Shao et al., 2017), to regulate protein translation (Sobala and Hutvagner, 2013), various cellular activities (Maute et al., 2013), to mediate the immune system (Zhang et al., 2014), and the response under stress conditions (Gebetsberger et al., 2017). Notably, some tsRNAs are known to directly bind to mRNA targets in a manner similar to the canonical miRNAs (Huang et al., 2017; Zhou et al., 2017). These tsRNAs are physically related to Argonaute (AGO) proteins (Ago1–4), as stated in reference (Kumar et al., 2014), indicating that a tsRNA may contain multiple mRNA-binding sites and may have adsorptive and suppressive effects on the target genes. Based on this function, using bioinformatic tools, potential target-binding mRNAs for four candidate tsRNA were calculated and filtered to construct tsRNA/mRNA interactions for bioinformatic analysis. Next, using GO and KEGG pathway analysis, we found that the target mRNAs are involved in various biological processes, intracellular parts, and binding as well as cellular signaling pathways. As an example, the regulation of the macromolecular metabolic process was identified to be one of the most significantly enriched and most meaningful terms of biological processes, which was confirmed to closely correlate to the pathophysiological process after SCI (Kumar et al., 2015; Shakhbazau et al., 2015). In the KEGG pathway, the MAPK and neurotrophin signaling pathways were the top two signaling pathways affected by the candidate tsRNA-mRNA axes. Previous studies (Zhang et al., 2015; Hodgetts and Harvey, 2017; Fu et al., 2018) have explored the relationships between these pathways and SCI, indicating that MAPK-related molecules regulate the neuronal survival, synaptic function, and neurotransmitter release, and elicit the plasticity and growth of axons within the central nervous system after SCI. Additionally, another report (Keefe et al., 2017) described that the neurotrophins, including NGF, BDNF, and NT-3, targeted specific populations of neurons *via* their effects on the populations of neurons within diverse spinal tracts.

Finally, using PCR, we found that the four candidate tsRNAs and BDNF, one of the neurotrophins predicted by the bioinformatic analysis as a target gene, had opposite alterations of the expression level, which indirectly suggests that these specific tsRNAs could inhibit the BDNF very likely *via* complementary base pairing. In addition, using the luciferase reporter assay we identified one candidate tsRNA, tiRNA-Gly-GCC-001, directly binding to BDNF, which indicates that tiRNA-Gly-GCC-001 might be involved in the MAPK and neurotrophin pathways *via* targeting the key gene, BDNF, thereby regulating the pathophysiological process after SCI.

However, several questions remain to be answered. First and most importantly, the reader should bear in mind that this study has finished the expression profile and functional prediction; thus, our future work is to identify the functions of the candidate tsRNAs *in vitro* and *in vivo*. Furthermore, it was beyond the scope of this study to examine the potential effect of different time points on the possible dynamic changes in the tsRNA expression patterns in the contused spinal cords of rats with SCI. Finally, we strongly recommend that future research should focus on different animal models and types of SCI to study the expression and functions of tsRNAs.

## Data Availability Statement

The raw data supporting the conclusions of this article will be made available by the authors, without undue reservation, to any qualified researcher.

## Ethics Statement

The animal study was reviewed and approved by the Institutional Animal Care and Use Committee of Capital Medical University.

## Author Contributions

CQ and HF performed the research. J-JL and FG designed the research study. CZ, XZ and Y-CS contributed essential reagents or tools. YL, D-GY and L-JD analyzed the data. CQ wrote the article. M-LY revised the article. All authors read and approved the final manuscript.

## Conflict of Interest

The authors declare that the research was conducted in the absence of any commercial or financial relationships that could be construed as a potential conflict of interest.

## Footnotes

^1^http://www.targetscan.org

^2^http://www.microrna.org

^3^http://www.mirdb.org

^4^http://www.geneontology.org

^5^www.genome.jp/kegg

## Abbreviations

SCI: spinal cord injury
tRNA: transfer RNA
tsRNA: tRNA-derived small RNA
qRT-PCR: quantitative reverse transcription-polymerase chain reaction
MAPK: mitogen-activated protein kinase
BDNF: brain-derived neurotrophic factor
ncRNAs: noncoding RNAs
lncRNAs: long non-coding RNAs
miRNAs: microRNAs
circRNAs: circular RNAs
mRNAs: messenger RNAs
tRFs: tRNA-derived fragments
tiRNAs: tRNA-derived stress-induced RNAs
FC: fold change
CPMs: counts per million
GO: Gene Ontology
KEGG: Kyoto Encyclopedia of Genes and Genomes database
FC: fold change.
