# Supplementary material for: Differential Expression Profiles and Functional Prediction of tRNA-Derived Small RNAs in Rats After Traumatic Spinal Cord Injury
Source: Front Mol Neurosci. 2020 Jan 10;12:326. doi: 10.3389/fnmol.2019.00326 (PMC6968126; doi:10.3389/fnmol.2019.00326)
Supplement: Supplementary file 1 [file Table_1.DOCX]

Supplementary Material

**Differential Expression Profiles and Functional Predication of tRNA-derived Small RNAs in Rats After Traumatic Spinal Cord Injury**

**Chuan Qin^1,2,3,4,5,#^, Hao Feng ^1,2,3,4,5,#^, Chao Zhang ^1,2,3,4,5^, Xin Zhang ^1,2,3,4,5^, Yi Liu ^1,2,3,4,5^, De-Gang Yang ^1,2,3,4,5^, Liang-Jie Du^1,2,3,4,5^, Ying-Chun Sun ^1,2,3,4,5^, Ming-Liang Yang^1,2,3,4,5^,Feng Gao^1,2,3,4,5, *^ Jian-Jun Li^1,2,3,4,5, *^**

*** Correspondence:**Dr. Jian-Jun Li, crrclijj@163.com and Feng Gao, [gaofeng5960@126.com](mailto:gaofeng5960@126.com).

^#^These authors contributed equally to this work

**Supplementary Figure 1. tsRNA-seq quality score plot for sham group.** The position in the read is plotted on the X-axis and the Q value is plotted on the Y-axis. The red line is the median Q score, and the blue line is the mean Q score. The boxplot represents the inter-quartile range, while the whiskers represent the 10% and 90% points. A Q score above 30 (>99.9% correct) is considered high quality data. (A) sample 1. (B) sample 2. (C) sample 3. (D) sample 4.


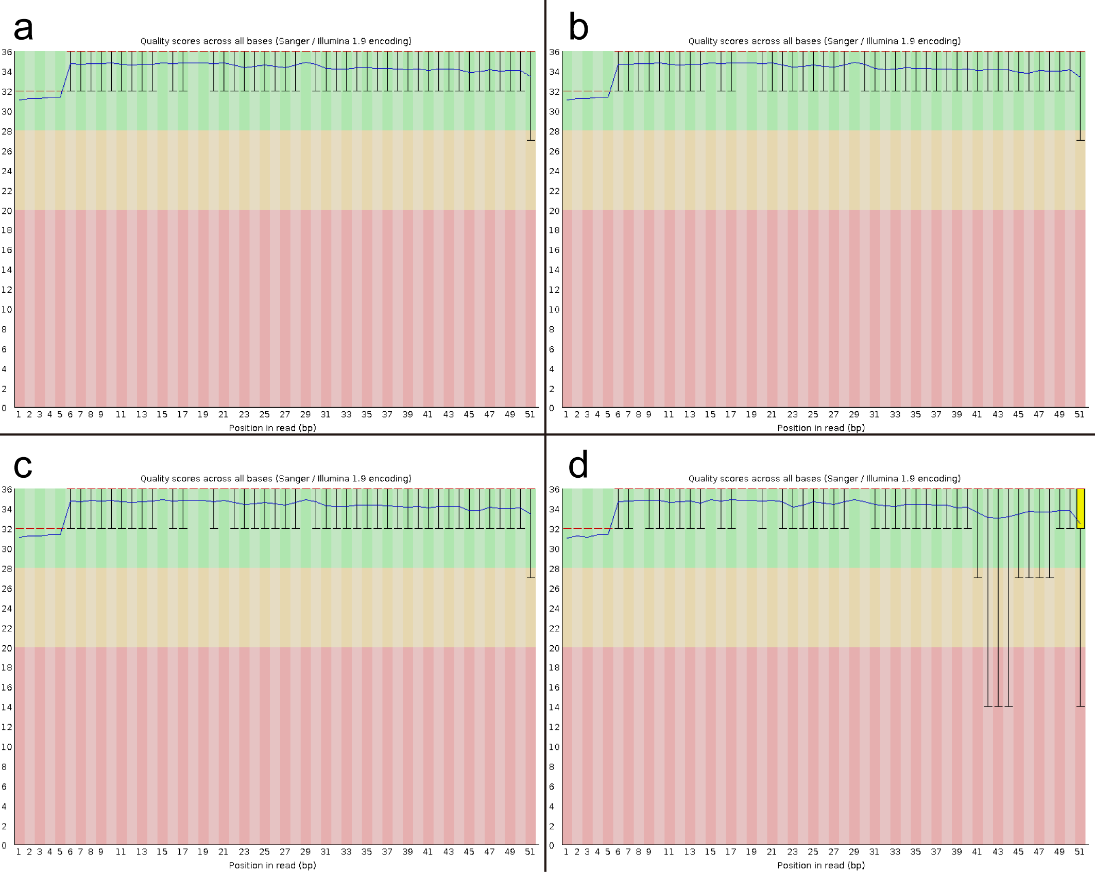


**Supplementary Figure 2.** **tsRNA-seq quality score plot for SCI group.** The position in the read is plotted on the X-axis and the Q value is plotted on the Y-axis. The red line is the median Q score, and the blue line is the mean Q score. The boxplot represents the inter-quartile range, while the whiskers represent the 10% and 90% points. A Q score above 30 (>99.9% correct) is considered high quality data. (A) sample 1. (B) sample 2. (C) sample 3. (D) sample 4.


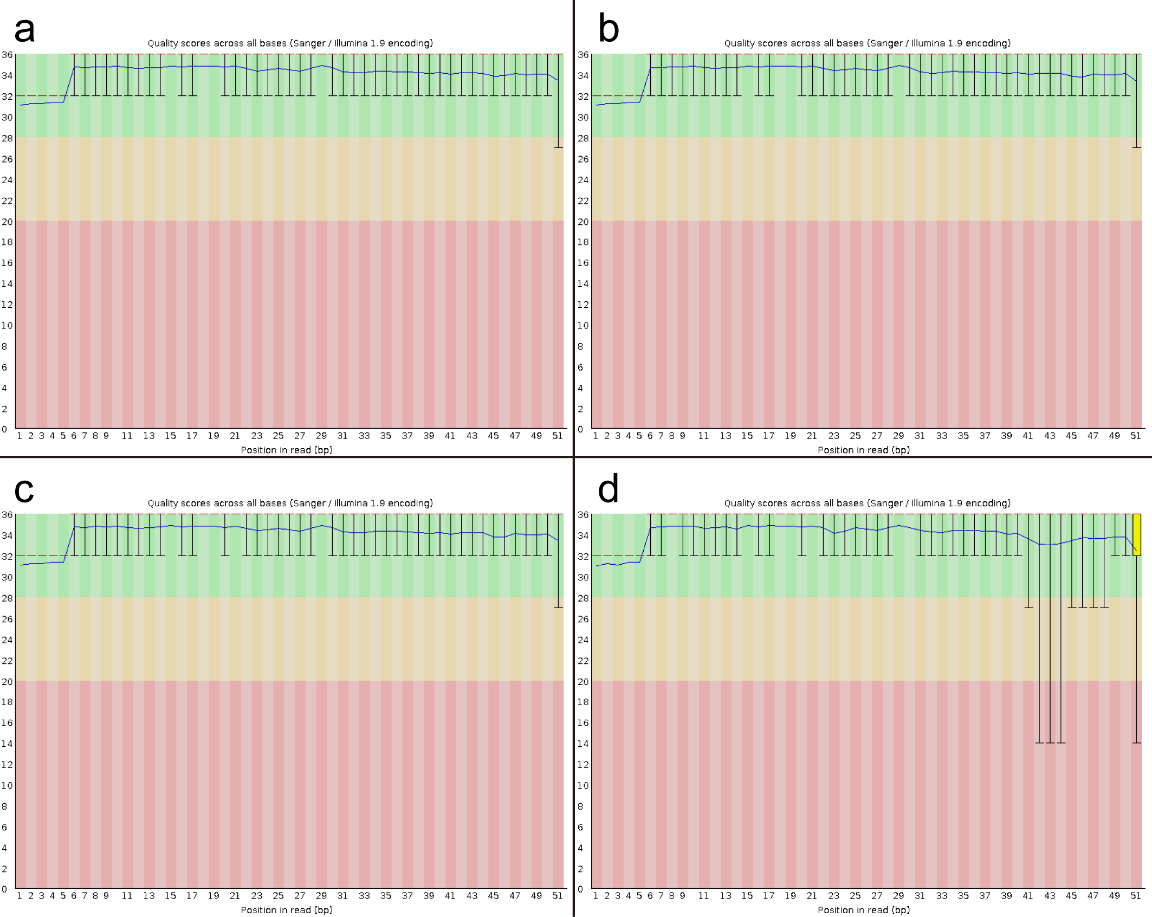


**Supplementary Figure 3. The enrichment score dot plot for cellular component of target mRNAs.** (A) tiRNA-Gly-GCC-001. (B) tRF-Gly-GCC-012. (C) tRF-Gly-GCC-013. (D) tRF-Gly-GCC-016.


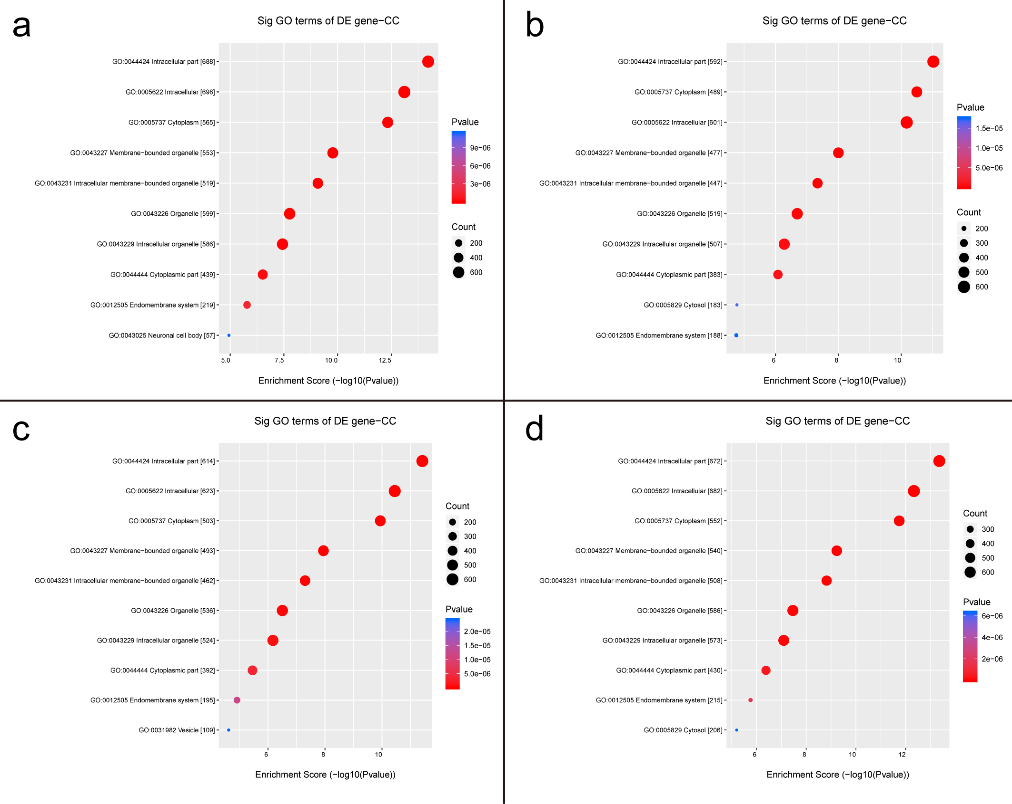


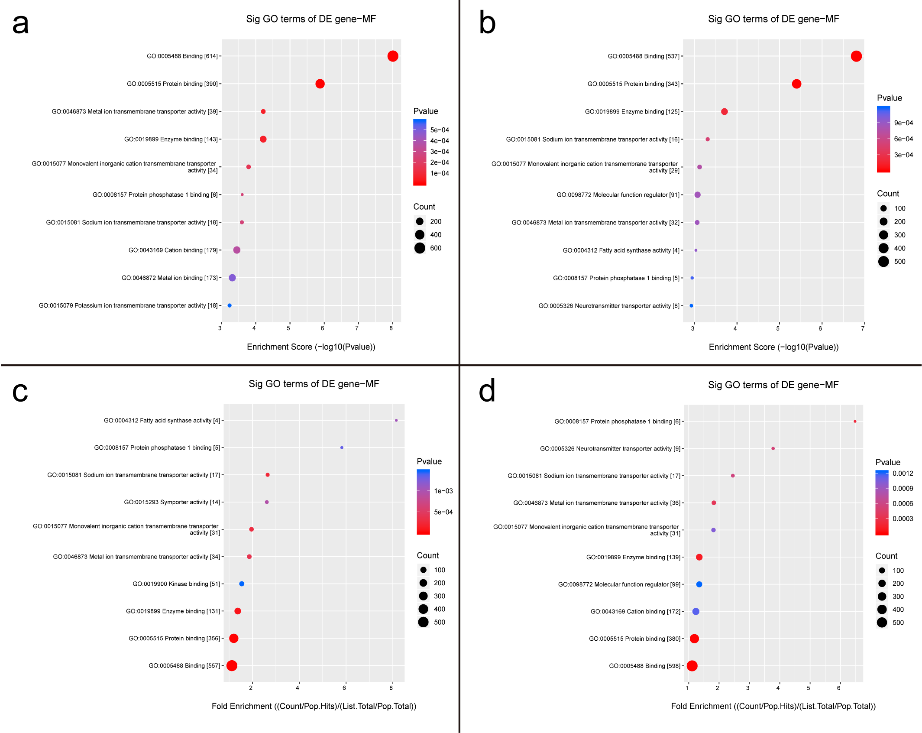
**Supplementary Figure 4.** **The enrichment score dot plot for molecular function of target mRNAs.** (A) tiRNA-Gly-GCC-001. (B) tRF-Gly-GCC-012. (C) tRF-Gly-GCC-013. (D) tRF-Gly-GCC-016.

**Supplementary Figure 5.** **The enrichment score dot plot for biological processes of target mRNAs.** (A) tiRNA-Gly-GCC-001. (B) tRF-Gly-GCC-012. (C) tRF-Gly-GCC-013. (D) tRF-Gly-GCC-016.


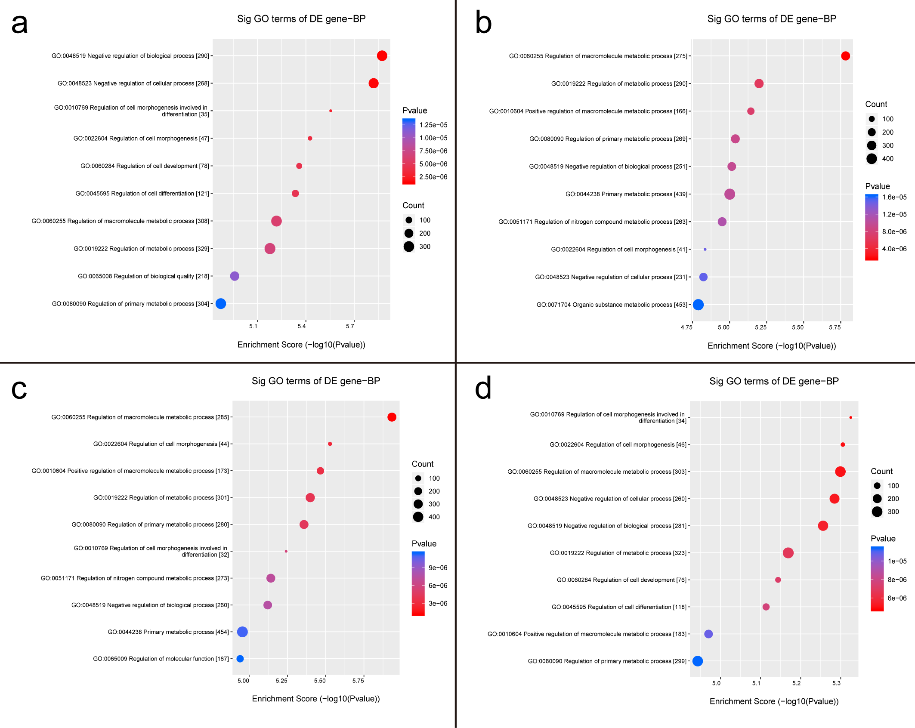


**Supplementary Figure 6. Target genes of the four tsRNAs in the MAPK signaling pathways predicted by KEGG.** Orange marked nodes indicate the target genes, and green nodes have no significance. (A) tiRNA-Gly-GCC-001. (B) tRF-Gly-GCC-012. (C) tRF-Gly-GCC-013. (D) tRF-Gly-GCC-016.


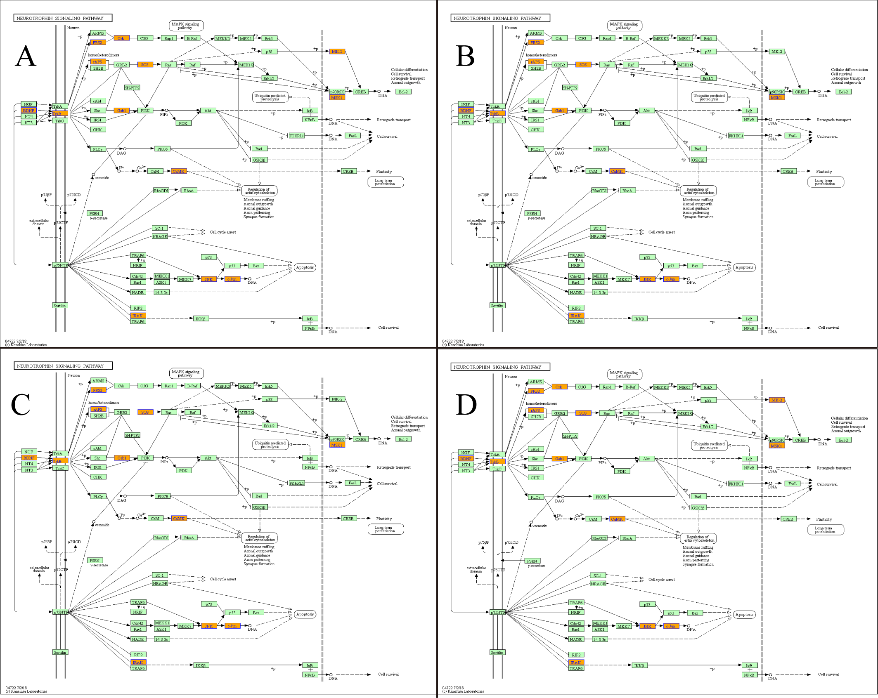


**Supplementary Figure 7. Target genes of the four tsRNAs in the neurotrophin signaling pathways predicted by KEGG.** Orange marked nodes indicate the target genes, and green nodes have no significance. (A) tiRNA-Gly-GCC-001. (B) tRF-Gly-GCC-012. (C) tRF-Gly-GCC-013. (D) tRF-Gly-GCC-016.


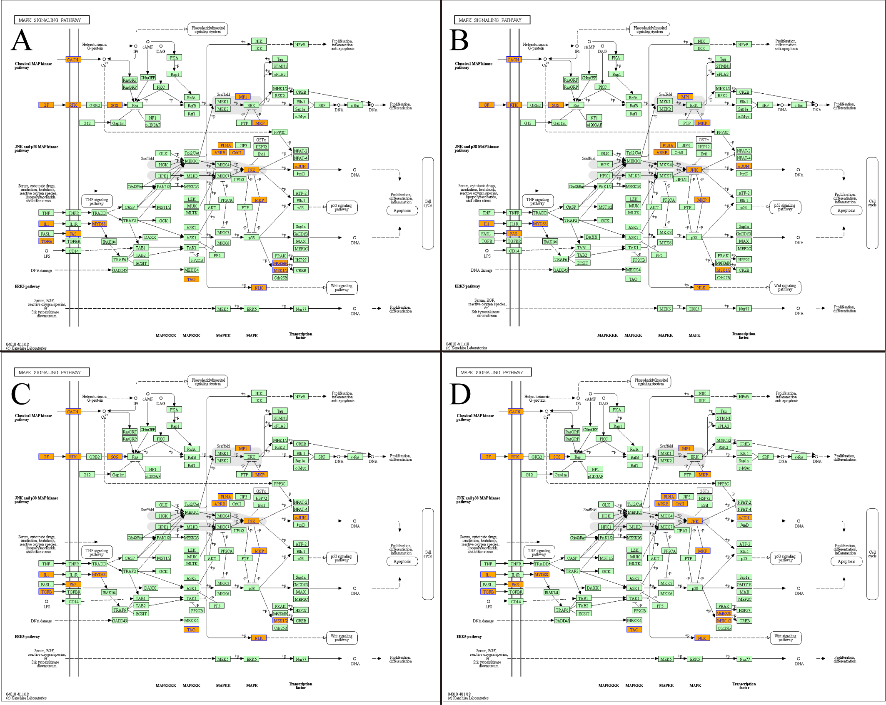


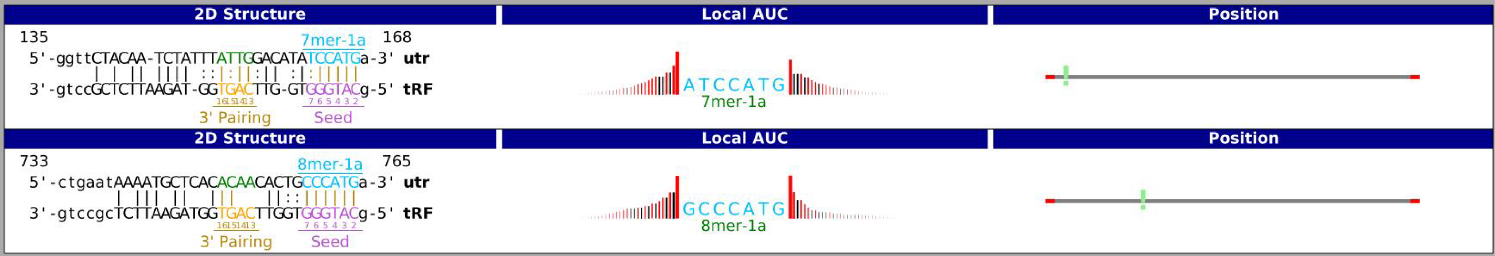
**Supplementary Figure 8.** The 2D structure and binding sites predicted by bioinformatics for BDNF and tiRNA-Gly-GCC-001.

**Supplementary Table 1. Quality score for each sample**

| **Sample** | **TotalRead** | **TotalBase** | **BaseQ30** | **BaseQ30 (%)** |
| --- | --- | --- | --- | --- |
| test-1 | 11718005 | 597618255 | 556071774 | 93.05 |
| test-2 | 8503022 | 433654122 | 402950178 | 92.92 |
| test-3 | 8816694 | 449651394 | 418370519 | 93.04 |
| test-4 | 10667327 | 544033677 | 505335723 | 92.89 |
| control-1 | 9389839 | 478881789 | 447828788 | 93.52 |
| control-2 | 7859905 | 400855155 | 374588918 | 93.45 |
| control-3 | 7799579 | 397778529 | 371901050 | 93.49 |
| control-4 | 10284968 | 524533368 | 487403189 | 92.92 |

Sample: Sample name

TotalRead: Raw sequencing reads after quality filtering

TotalBase: Number of bases after quality filtering

BaseQ30: Number of bases of Q score more than 30 after quality filtering

BaseQ30 (%): The proportion of bases (Q⩾30) number after quality filtering
